# Revealing Molecular Mechanisms of the Bioactive Saponins from Edible Root of *Platycodon grandiflorum* in Combating Obesity

**DOI:** 10.3390/plants13081123

**Published:** 2024-04-17

**Authors:** Bincheng Han, Jinhai Luo, Baojun Xu

**Affiliations:** Guangdong Provincial Key Laboratory IRADS and Department of Life Sciences, BNU-HKBU United International College, Zhuhai 519087, China; q030013010@mail.uic.edu.cn (B.H.); luojinhai@uic.edu.cn (J.L.)

**Keywords:** *Platycodon grandiflorum*, saponins, obesity, network pharmacology

## Abstract

Obesity has emerged as a significant health concern, as it is a disease linked to metabolic disorders in the body and is characterized by the excessive accumulation of lipids. As a plant-derived food, *Platycodon grandiflorum* (PG) was reported by many studies, indicating that the saponins from PG can improve obesity effectively. However, the anti-obesity saponins from PG and its anti-obesity mechanisms have not been fully identified. This study identified the active saponins and their molecular targets for treating obesity. The TCMSP database was used to obtain information on 18 saponins in PG. The anti-obesity target of the PG saponins was 115 targets and 44 core targets. GO and KEGG analyses using 44 core anti-obesity genes and targets of PG-active saponins screened from GeneCards, OMIM, Drugbank, and DisGeNet showed that the PI3K-Akt pathway, the JAK-STAT pathway, and the MAPK pathway were the major pathways involved in the anti-obesity effects of PG saponins. BIOVIA Discovery Studio Visualizer and AutoDock Vina were used to perform molecular docking and process the molecular docking results. The molecular docking results showed that the active saponins of PG could bind to the major therapeutic obesity targets to play an obesity-inhibitory role. The results of this study laid the foundation for further research on the anti-obesity saponins in PG and their anti-obesity mechanism and provided a new direction for the development of functional plant-derived food. This research studied the molecular mechanism of PG saponins combating obesity through various signaling pathways, and prosapogenin D can be used to develop as a new potential anti-obesity drug.

## 1. Introduction

In today’s society, obesity has dramatically increased to epidemic proportions and has become the leading cause of ill health [1]. Obesity, usually defined as an individual’s body mass index (BMI) ≥ 30 kg/m^2^, is caused by an imbalance between the body’s daily energy intake and energy expenditure; obesity has been recognized as a serious and rapidly escalating public health problem in the world [2,3]. Obesity is a chronic multi-system disease that is closely related to metabolic disorders in the body, which also increases psychological distress in obese patients and exacerbates the occurrence of obesity-related complications [4]. Obesity is strongly associated with increased blood pressure, diabetes, atherosclerosis, and cardiovascular disease, among other diseases caused by abnormal metabolism in the body. These diseases are mainly caused by eating excessive fat in the diet [5]. A clearer understanding of the impact of obesity and its complications on health is necessary to identify high-risk individuals and prioritize treatment [6]. Pre-diabetic patients can prevent and even reverse type 2 diabetes with early weight loss [7]. Obesity can now occur at any age and has a negative impact on the quality of life of both men and women [8].

Obesity is a serious health problem that is closely associated with metabolic disorders in the body [9]. In the body, an excessive accumulation of lipids, particularly in hepatocytes and adipocytes, is one of the main causes of this metabolic disorder. Understanding and intervening in lipid metabolism, especially controlling the proliferation of adipocytes, may be the key to combating obesity and its associated diseases [10]. It is crucial to comprehend why obesity is closely linked to health issues, as it can aid in the creation of successful prevention and treatment methods. Obesity is caused by the body’s impaired glucose and lipid metabolism, with insulin action primarily targeting hepatocytes and adipocytes [11]. If lipids accumulate in cells due to disturbed glucose–lipid metabolism, it can reduce insulin sensitivity in target cells like hepatocytes and adipocytes, leading to chronic metabolic diseases associated with insulin resistance [12]. Therefore, inhibiting adipocyte differentiation and intracellular fat proliferation to ameliorate insulin resistance is an effective way to prevent or treat fat production in the body.

Currently, popular anti-obesity drugs include sympathomimetic drugs such as phentermine, diethylpropion, sibutramine, and orlistat, but taking them can cause cardiovascular diseases such as heart valve damage, pulmonary hypertension, myocardial infarction, diarrhea, flatulence, incontinence, and dyspepsia, as well as liver damage [13]. In contrast, natural organic compounds have been used in plant-derived food for a very long history with consistent safety and high efficacy [14]. To date, many plant-derived foods can be used in the daily diet and can act as obesity inhibitors [15]. For example, the saponin components of PG can be used to treat obesity. Several studies have shown that diets rich in saponins from PG can reduce systemic cholesterol levels and inhibit obesity [16]. Saponins in PG can lower the rate of bile acid reuptake and the content of serum cholesterol in the blood.

*Platycodon grandiflorum* (PG) is a monotypic species of the genus *Platycodon* in the family Campanulaceae, which commonly grows in China, North Korea, Republic of Korea, Japan, and eastern Siberia area in Russia [17,18]. The fresh or dry root of PG has high nutritional value; it is a popular appetizer vegetable in Northeast China and the Korean peninsula. The roots of PG are called Platycodi Radix. There is a mixture of various compounds containing many types and amounts of bioactive compounds in PG, including flavonoids, polyphenols, and saponins with some protective effects, such as antioxidant, anti-inflammatory, hypolipidaemic, anticancer, and hepatoprotective effects. The potential new lead compound in PG is saponin [19], and more specifically, PG saponin can inhibit obesity in vivo and in vitro and is used to treat obesity in clinics [20,21]. The PG saponin showed anti-obesity effects and lowered systemic cholesterol in the high-fat-diet-induced obese mice [22,23]. There is research about the active compounds in PG to improve and regulate the mechanism of action of obesity and glucose–lipid metabolism that has received more attention, but the mechanism of PG lipid-lowering and anti-obesity action and the effective compounds of the anti-obesity is still not completely clear.

Network pharmacology can be effective in understanding the relationship between multiple non-specific compounds and multiple genetic proteins [24]. Network pharmacology can also help researchers study the molecular mechanism of plant-derived food by analyzing compounds in the plant and identifying new mechanisms for treating various diseases [25]. There are effective compounds and molecular mechanisms of Cnidii Fructus for the treatment of atopic dermatitis that can be identified through network pharmacology [26]. Therefore, using network pharmacology to screen saponins in PG and the targets of action of PG, as well as using target construction, the GO and KEGG pathways can be analyzed to construct a network of saponin constituents in PG related to anti-obesity proteins and predict feasible manipulations of PG to fight obesity.

## 2. Results

### 2.1. The Active Saponins of PG

The screening for active saponins in PG are considered active with an OB of at least 30% and DL score of at least 0.18. Eighteen saponin constituents were identified in PG and are shown in Table 1, including platycodin A, platycodin C, platycodin D, deapioplatycodin D, platycodin D2, platycodin D3, deapioplatycodin D3, deapioplatycoside E, platyconic acid B lactone, 3″-O-acetylplatyconic acid A, prosapogenin D, polygalacin D, polygalacin D3, platycoside E, polygalacin D2, dimethyl 2-O-methyl-3-O-a-D-glucopyranosyl platycogenate A, platycodin V, and polygalacin XI.

### 2.2. Targets and Statistics on Obesity-Related Genes of PG Saponins

**Figure 1:**
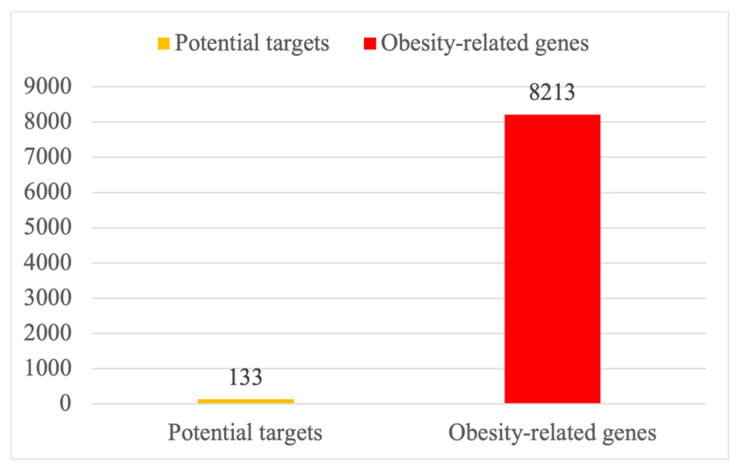
A total of 133 predicted potential targets of PG saponins and 8213 obesity-related genes.

To obtain direct targets for 18 saponins of PG, target prediction was performed using SwissTargetPrediction, similarity ensemble approach (SEA), and TCMSP databases. After further removal of redundancy and the deletion of duplicate targets, the summary of 133 possible targets was discerned [30,31,32]. These 133 predicted potential targets were further analyzed (Figure 1).

The number of 8213 obesity-related genes was gained using a search for the keyword “obesity” in the GeneCards database [33] (Figure 1).

**Figure 2:**
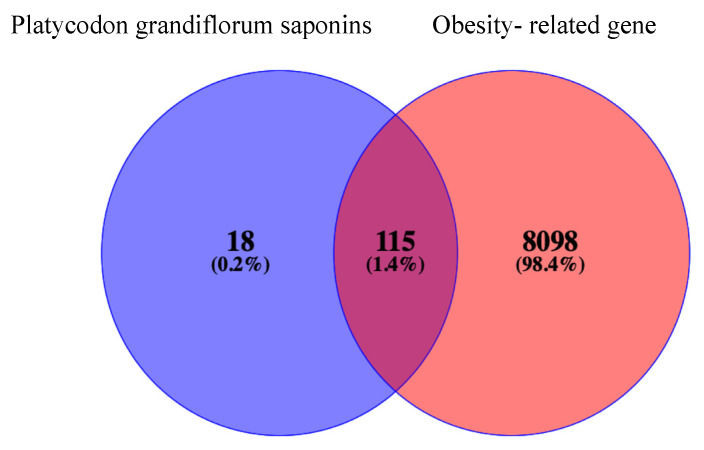
The Venn diagram of intersection of PG saponins and obesity-related genes.

The intersection between obesity-related targets and active saponins in PG was identified through using the Venny 2.1.0 online system. The results of this analysis are shown in Figure 2. A total of 115 saponin targets were identified as intersecting with obesity disease genes [34].

### 2.3. Protein–Protein Interaction (PPI) Analysis of PG Saponins

**Figure 3:**
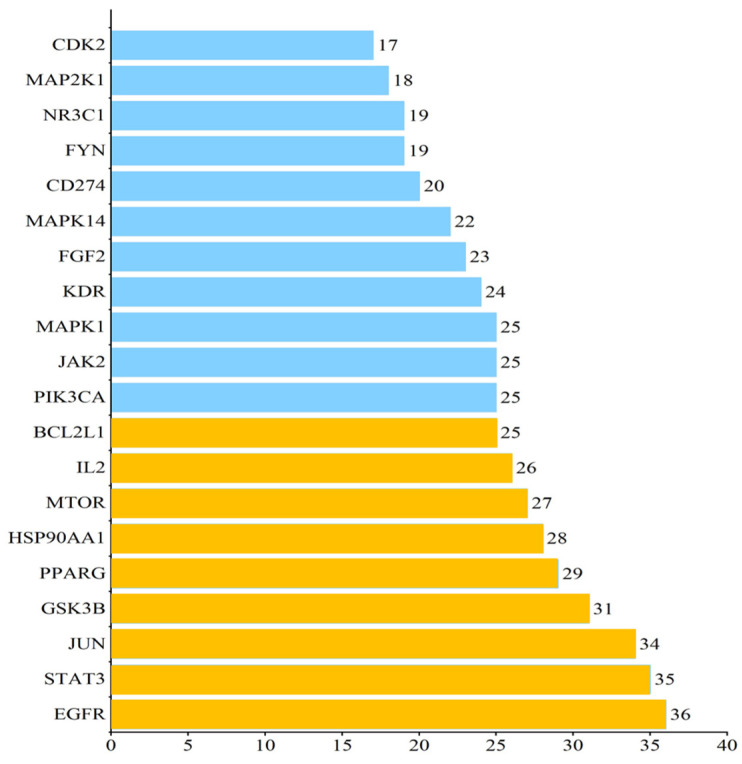
Top eighteen targets from PG ranked by the degree of potential targets related to obesity. The yellow bars stand for the top nine PG targets with DC value greater than or equal to 25, which is used for molecular docking. The blue bars stand for the PG targets with DC value less than or equal to 25.

The eighteen targets from PG were ranked in order of the degree of potential targets related to obesity they contain, as shown in Figure 3. The highest-ranked target was EGFR, which appeared in 36 forms at an intersection of 115 PG-active saponins and possible anti-obesity targets, which may be a core anti-obesity target. In addition, the top nine targets with a degree value greater than 25 were taken for subsequent molecular docking validation.

**Figure 4:**
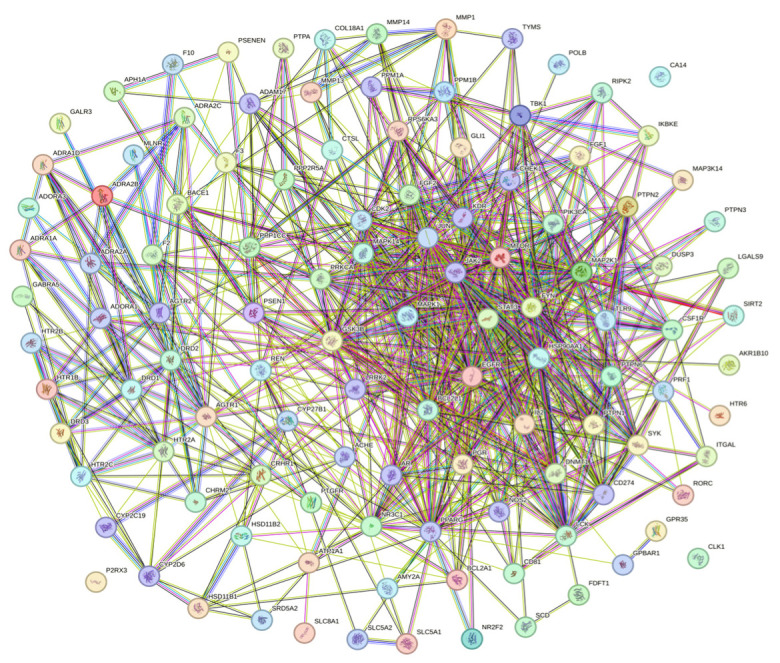
PPI network of 115 PG saponins potential anti-obesity targets.

**Figure 5:**
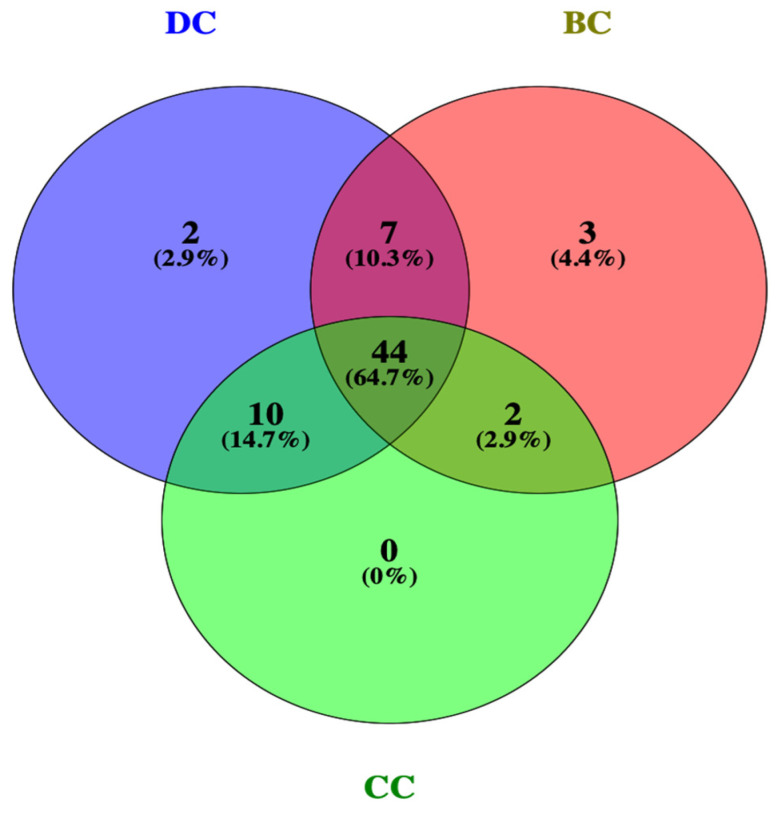
DC, BC, and CC screen 44 potential anti-obesity targets. DC, degree centrality; BC, betweenness centrality; CC, closeness centrality.

A total of 115 potential anti-obesity targets of PG saponins were imported into STRING 11.5, and a PPI network was established (Figure 4) [35]. The potential anti-obesity targets of 115 PG saponins were screened by the CytoNCA web tool (https://cytoscape.org/ (accessed on 18 August 2023)) for three topologies of degree centrality (DC), betweenness centrality (BC), and closeness centrality (CC), and the screened more core of 44 potential anti-obesity core targets of PG saponins were obtained as shown in Figure 5, and the structures were displayed using Venny 2.1.0 tool [34,36].

**Figure 6:**
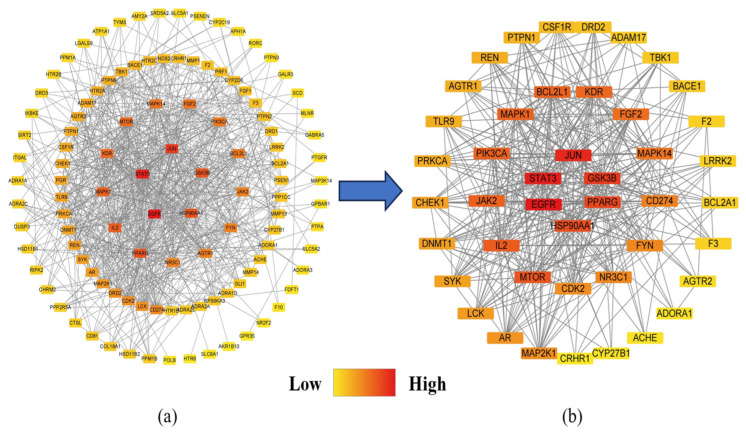
PPI analyses of the PG saponins before and after screening for potential targets. (**a**) PPI network of all 115 targets of PG saponins. (**b**) PPI network of 44 screened targets based on the median value of BC, CC and DC.

**Figure 7:**
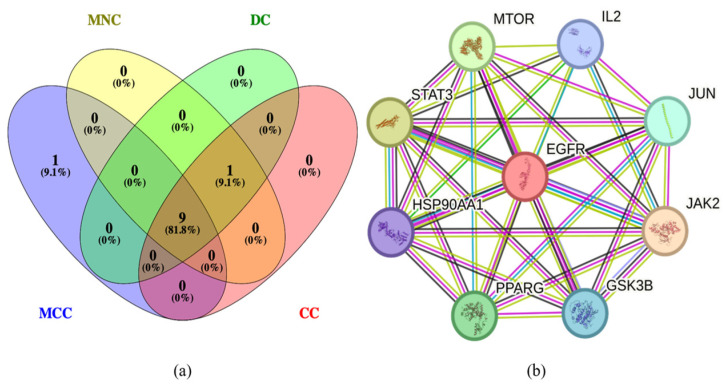
MNC, MCC, DC, and CC screening core targets (**a**) and PPI network of the nine anti-obesity core targets (**b**). MNC, maximum neighborhood component; MCC, maximal clique centrality; DC, degree centrality; CC, closeness centrality.

The results of the PPI analyses of the PG saponins before and after screening for potential targets for obesity were exported as simple text data format files and then imported into Cytoscape 3.9.0 software to establish the PPI network shown in Figure 6a,b, Figure 6a is sorting of 115 targets and Figure 6b is sorting of the filtered 44 targets. To describe this network graph, the nodes are represented by colored circles, with each color indicating its degree from the lowest (yellow) to the highest (red). Meanwhile, nine anti-obesity central targets, i.e., EGFR, ESK3B, PPARG, HSP90AA1, STAT3, MTOR, IL2, JUN, and JAK2 were screened using MNC, MCC, DC, and CC in the CytoHubba network tool (Figure 7a), and these central targets are selected for molecular docking later [36]. The screened structures and the PPI network analysis of the nine anti-obesity central targets are shown in Figure 7b.

### 2.4. The Network of Active Saponins and Anti-Obesity Targets of PG Saponins

**Figure 8:**
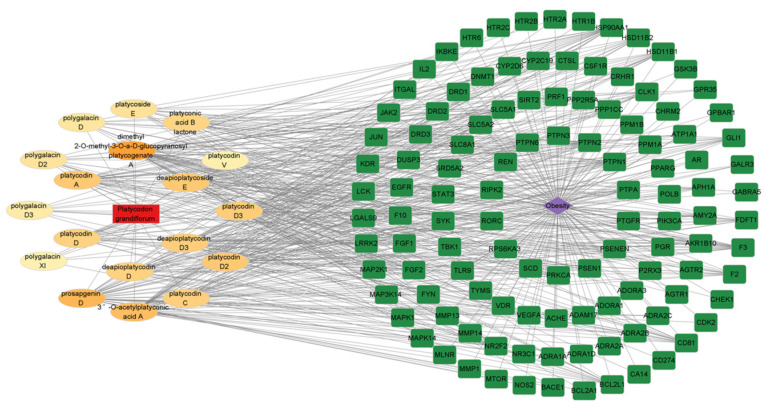
Drug–Compounds–Targets–Disease network of PG saponins and obesity.

The information presented in the diagram depicted in Figure 8, a network was generated using Cytoscape 3.9.0, which displays 115 targets of 18 PG saponin-active compounds. These compounds are known to act against obesity, as described in the PPI analysis part. Additionally, a network of hubs was constructed between 115 anti-obesity targets and 18 PG saponin active compounds. All 18 PG saponin active compounds acted on the 115 anti-obesity targets.

The Drug–Compounds–Targets–Disease network shows that each of the saponins in PG can interact with multiple targets, while several sides of the PG saponins can relevantly interact with a single target. This result demonstrates that the saponins are quite complex in PG interacting with multiple core anti-obesity targets [37]. Interactions between 18 active saponins from PG and 44 anti-obesity targets from obesity were explained.

**Figure 9:**
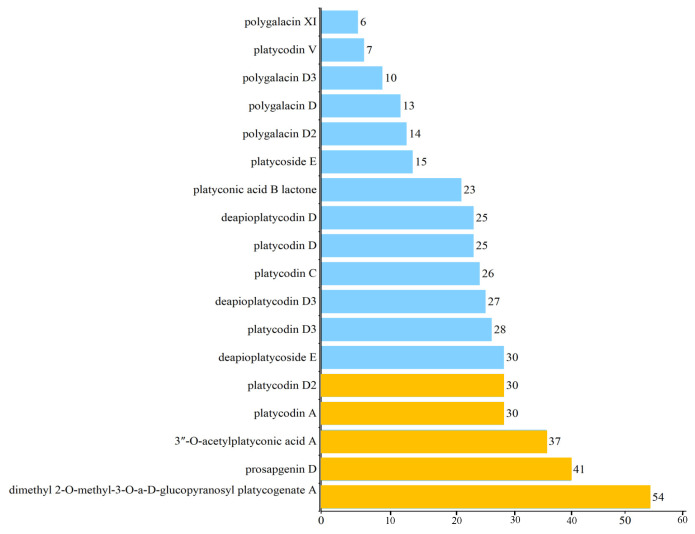
Active saponins from PG ranked by degree. The yellow bars stand for PG saponins with DC value greater than or equal to 30, which is used for molecular docking. The blue bars stand for PG saponins with DC value less than or equal to 30.

The DC values of 18 PG saponins in the network of active saponins and anti-obesity targets of PG saponins are shown in Figure 9. The top ten PG saponins (key compounds with more than 25 targets each) are dimethyl 2-O-methyl-3-O-a-D-glucopyranosyl platycogenate A, prosapogenin D, 3″-O-acetylplatyconic acid A, platycodin A, platycodin D2, deapioplatycoside E, platycodin D3, deapioplatycodin D3, platycodin C, and platycodin D. For subsequent molecular docking validation, these saponins were selected to be tested against the top nine ranked targets.

### 2.5. GO Enrichment and KEGG Signaling Pathway Analysis of PG Saponins

**Figure 10:**
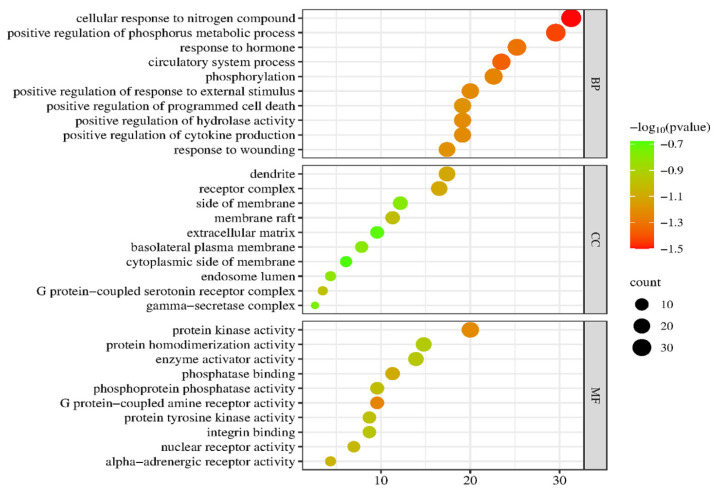
GO enrichment analysis of PG saponins. BP, biological process; CC, cellular component; MF, molecular function.

The GO enrichment analysis was performed on previously derived 44 anti-obesity core targets. Figure 10 shows the top 10 enrichment terms for each of the 44 anti-obesity core targets: BP, CC, and MF. The GO enrichment analysis results indicated that anti-obesity targets of PG saponins were involved in various biological processes.

**Figure 11:**
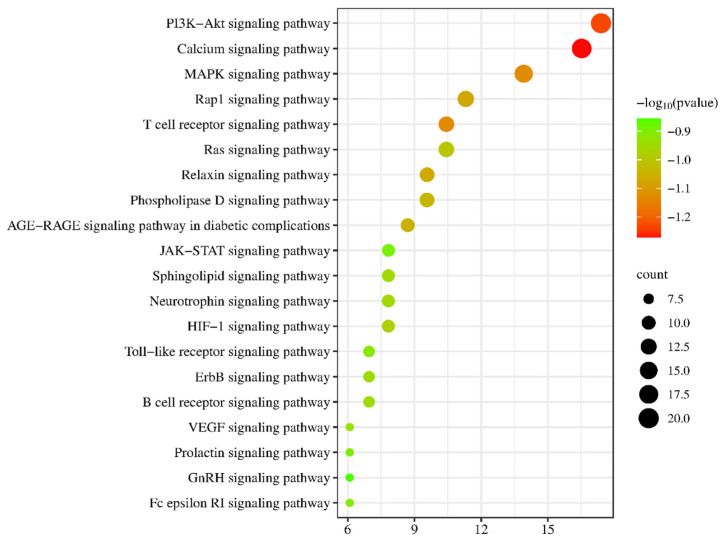
The number of top 20 signaling pathways related to PG saponins.

KEGG signaling pathways were expressed to infer the molecular mechanism of anti-obesity of active saponin components in PG, which was necessary. As shown in the figure, after uploading 44 PG anti-obesity core targets to the Metascape, the top 20 KEGG signaling pathways were listed in Figure 11. The KEGG signaling pathway enrichment analysis listed that the top three signaling pathways in KEGG molecules are the PI3K-Akt signaling pathway, the Calcium signaling pathway, and the MAPK signaling pathway. PG saponins mainly regulate these three signaling pathways to anti-obesity targets. PG’s active saponins may impact obesity’s molecular mechanism [38].

### 2.6. Molecular Docking

**Figure 12:**
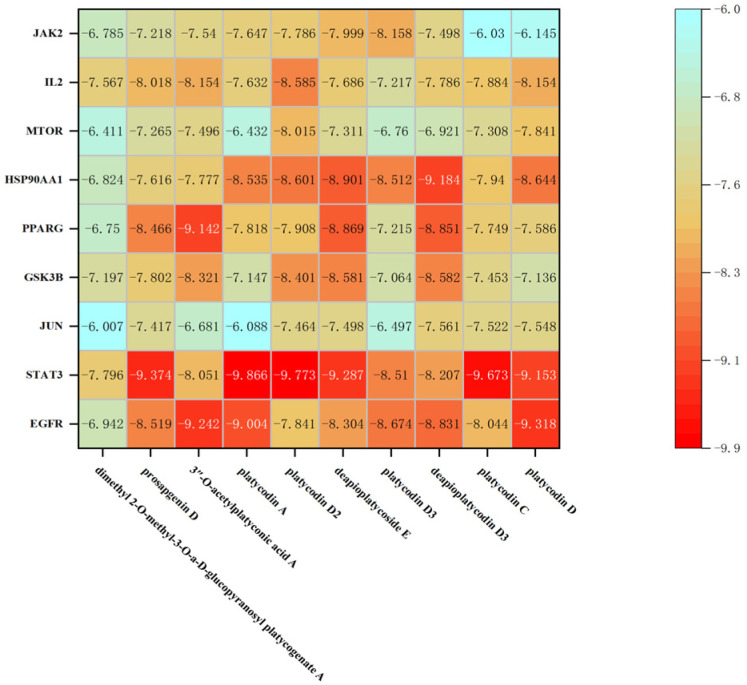
The binding energy network between saponins and anti-obesity targets.

**Figure 13:**
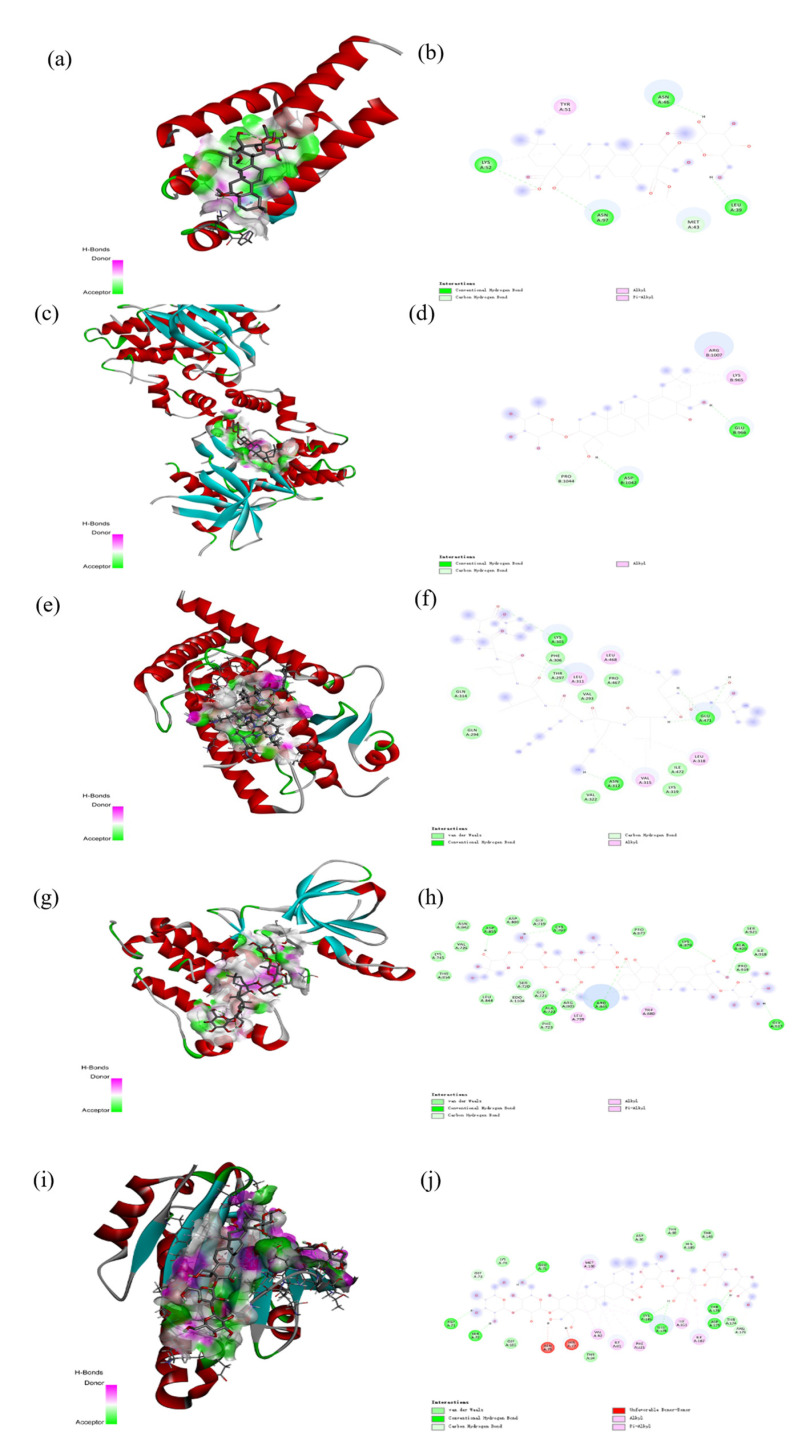
The 3D (**a**) and 2D (**b**) structures of molecular docking between dimethyl 2-O-methyl-3-O-a-D-glucopyranosyl platycogenate A and IL2. The 3D (**c**) and 2D (**d**) structures of molecular docking between prosapgenin D and STAT3. The 3D (**e**) and 2D (**f**) structures of molecular docking between 3″-O-acetylplatyconic acid A and PPARG. The 3D (**g**) and 2D (**h**) structures of molecular docking between platycodin A and EGFR. The 3D (**i**) and 2D (**j**) structures of molecular docking between platycodin D2 and HSP90AA1.

**Figure 14:**
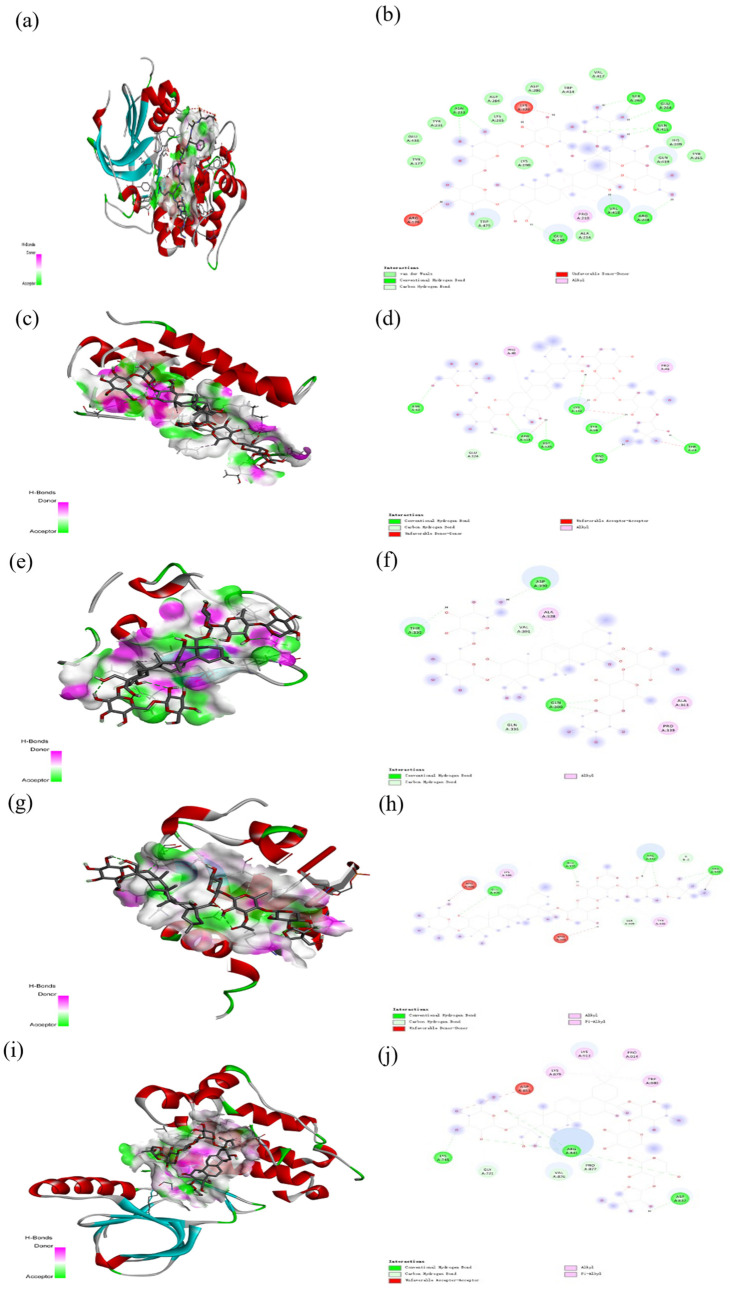
The 3D (**a**) and 2D (**b**) structures of molecular docking between deapioplatycoside E and GSK3B. The 3D (**c**) and 2D (**d**) structures of molecular docking between platycodin D3 and JAK2. The 3D (**e**) and 2D (**f**) structures of molecular docking between deapioplatycodin D3 and JUN. The 3D (**g**) and 2D (**h**) structures of molecular docking between platycodin C and MTOR. The 3D (**i**) and 2D (**j**) structures of molecular docking between platycodin D and EGFR.

As shown in Figure 12, Figure 13 and Figure 14, molecular docking was performed for the major PG active saponins with nine central anti-obesity targets of PG saponins. The interaction results of various saponins with different proteins were analyzed in 3D and 2D structures.

The binding energy shows the affinity between the saponin compound and the protein. The lower energy indicates the better binding of the receptor protein [39]. The results of the docking experiment showed that core active saponins from PG bind to nine central anti-obesity targets. These findings suggest that the major saponins of PG can play a direct role in central anti-obesity targets, such as EGFR, ESK3B, PPARG, HSP90AA1, STAT3, MTOR, IL2, JUN, and JAK2, which are related to signaling pathway in molecular docking. The verification of the interaction between the saponin and the central targets can be analyzed by molecular docking in the study of Guo et al. and Gu et al. [40,41]. The molecular docking results of this study are consistent with those binds through network pharmacology, providing additional support to the results in this research.

### 2.7. ADMET Analysis

Using a web-based method for ADMET analysis is an important tool in discovering and developing new medicines [42]. ADME analysis and small molecule toxicity analysis were performed using the SwissADME database and the ProTox II—Prediction of Toxicity of Chemicals database [43]. The results showed that the five active saponins derived from PR with DC values greater than 30 had good pharmacokinetic properties (Table 2). The pharmacokinetic profiles of all five active saponins in various predictive models include gastrointestinal absorption, blood–brain barrier penetration, P-glycoprotein substrate, cytochrome P450 inhibitor, and skin penetration (Table 2).

In ADME analyses, the results for the first four saponins were consistent, with low expression in GI absorption, No in BBB permeant expression, all Yes in P-gp substrate expression, and No in CYP1A2, CYP2C9, CYP2C19, CYP2D6, and CYP3A4 inhibitor detection. Because the predicted LD50 value of dimethyl 2-O-methyl-3-O-a-D-glucopyranosyl platycogenate A is 1500 mg/kg, which is higher than that of the other four active saponins at 4000 mg/kg, and among the four remaining active saponins, only prosapgenin D had no cytotoxicity, the three remaining active saponins, 3″-O-acetylplatyconic acid A, platycodin A, and platycodin D2, had higher cytotoxicity than the other four active saponins. The confidence level of the cytotoxicity test was 0.70, and all of them were active, so prosapgenin D was again selected for KEGG analysis and molecular docking.

### 2.8. The KEGG Analysis of Prosapogenin D

**Figure 15:**
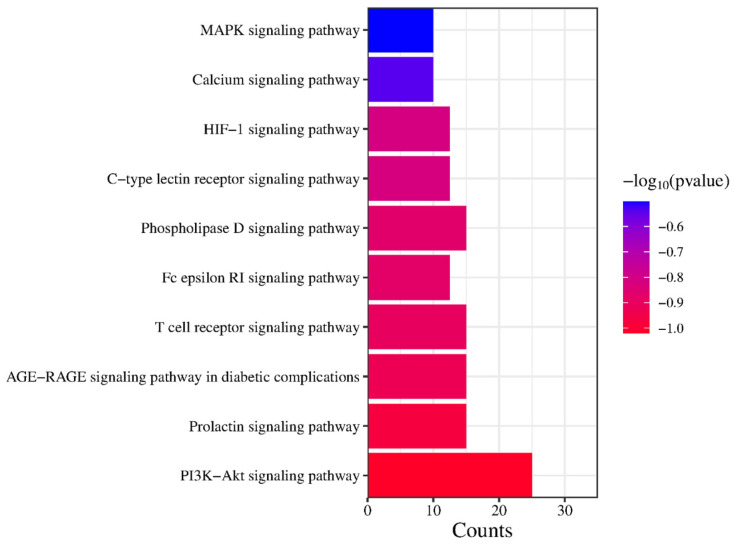
The number of top 10 signaling pathways related to prosapogenin D.

As with the purpose of the KEGG analysis described above, the KEGG analysis of prosapogenin D allowed a more detailed inference of the molecular mechanisms of the anti-obesity effects of PG. The anti-obesity targets of prosapogenin D were sent to the DAVID tool, which showed the ranking top ten KEGG pathways of prosapogenin D; the KEGG signaling pathway enrichment results demonstrated that similar to the results of all active saponins in PG, anti-obesity targets of prosapogenin D were mainly included PI3K-Akt signaling pathway, while Calcium and MAPK signaling pathway also appeared in the KEGG analysis results of prosapogenin D (Figure 15), which also proved that PG saponin prosapogenin D played an anti-obesity role, validating the plausibility of the experiment. These signaling pathways may play a part in treating obesity by the molecular mechanism underlying Prosaponinogen D. Prosapogenin D was also selected for molecular docking with regulatory proteins in the PI3K-Akt signaling pathway to provide more in-depth research in this study.

### 2.9. The Active Saponins of PG

**Figure 16:**
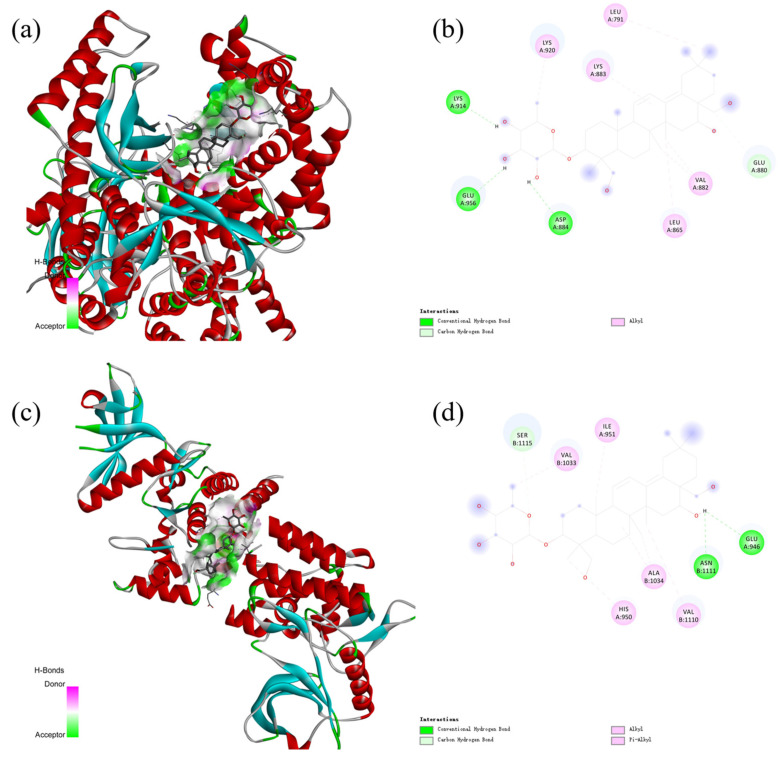
The 3D (**a**) and 2D (**b**) structures of molecular docking between prosapogenin D and PI3K. The 3D (**c**) and 2D (**d**) structures of molecular docking between prosapogenin D and Akt.

Central anti-obesity targets in the PI3K-Akt signaling pathway include PI3K and AKT. For docking studies, obesity-inhibiting crystal structures of the PI3K-Akt pathway (PDB ID: 4FA6 and 4GV1) were obtained from the PDB for molecular docking studies [43,44,45]. Table 3 and Figure 16 show the structures resulting from the molecular docking of prosapogenin D to the two central anti-obesity targets of the PI3K-Akt signaling pathways.

Prosapogenin D was docked with various proteins; there are many different types of interactions in the results of molecular docking. Binding capacity in molecular docking of prosapogenin D and the two regulatory proteins of the PI3K-Akt signaling pathway were much less than −6.0, prosapogenin D and PI3K (−8.139 KJ/mol) and prosapogenin and Akt (−8.494 KJ/mol), which indicated that prosapogenin D could play an anti-obesity effect by using the PI3K-Akt signaling pathway very well.

## 3. Discussion

Past research has mainly focused on the obesity suppression characteristics of saponin platycodin D, exploring the different pathways by which platycodin D inhibits and resists obesity caused by different causes, whereas the present study found that PG contains other active saponins in addition to platycodin D. The aim of this research was to explore if the other active saponins in PG have anti-obesity effects and if, by their potential molecular mechanisms, they treat can obesity.

The present study focuses on the molecular mechanisms of PG-active saponins in the fight against obesity. The pathogenesis of obesity has many mechanisms, and a variety of signaling pathways may simultaneously contribute to the production of body fat or inhibit the production of obesity and participate in the regulation of obesity. For example, the MAPK signaling pathway controls adipogenesis by regulating appetite and homeostasis of fat and glucose in the human body, and the PI3K-Akt signaling pathway has an anti-obesity effect by reducing appetite through the central nervous system and peripheral tissues to control obesity [46]. These pathways can also play roles in regulating and inhibiting adipogenesis together [47]. PG saponins can also inhibit obesity in clinics through the interaction between viscera [21].

In this study, 18 active saponins with OB of at least 30% and a DL value of at least 0.18 were screened for further research. It was found that these 18 active saponins have multi-targeting effects, and they can work together to perform anti-obesity functions with multiple target proteins to regulate obesity by different signaling pathways. Network pharmacology results showed that the top 10 PG active saponins could play a role on 25 or more core anti-obesity targets in terms of interaction with anti-obesity targets; they are as follows: dimethyl 2-O-methyl-3-O-a-D-glucopyranosyl platy-cogenate A, prosapogenin D, 3″-O-acetylplatyconic acid A, platycodin A, platycodin D2, deapioplatycoside E, platycodin D3, deapioplatycodin D3, platycodin C, and platycodin D. These 10 saponin-active compounds can be considered to be the most important anti-obesity saponins of PG. The OB values of all these saponins were bigger than 30, suggesting that oral administration of these saponins should be effective and have a positive anti-obesity effect [21]. In the traditional treatment of obesity, oral medication and/or external treatment modalities, such as acupuncture, are the commonly used therapeutic modalities for the treatment of obesity [48]. Further research is needed to determine if the active saponins in PG can be developed as effective treatments for obesity, whether administered orally or topically.

The results of PPI analysis indicated that many anti-obesity targets are involved in the anti-obesity process of PG-active saponins. The most involved anti-obesity proteins include EGFR, STAT3, JUN, GSK3B, PPARG, and HSP90AA1. The EGFR mediates the activation of adipose tissue macrophages for obesity resistance effects, the STAT3 can resist obesity caused by using too much fat by regulating adipose tissue T-cell populations, the JUN is involved in accelerating kinase-induced insulin resistance to obesity, the GSK3B enhances obesity, and the GSK3B enhances the therapeutic potential of adipose tissue macrophages for obesity by regulating specific biomarker proteins associated with obesity resistance and by shifting macrophage polarization and enhancing the therapeutic potential of adipose tissue macrophages [49,50,51,52]. Other genes may also be involved in different aspects of the anti-obesity regulatory process. For instance, the PPARG can regulate systemic insulin resistance, PPARG dysregulation will cause obesity, and HSP90AA1 can inhibit the reduction of adipogenesis through the in vivo pathway to trigger the anti-obesity effect [53,54].

GO enrichment analysis expressed that active saponins from PG combat obesity by affecting a variety of biological processes. Protein kinase activity inhibits obesity by modifying the activity of AMP kinase, which regulates the metabolism of fatty acids and glucose in the human body. The biological process of hormone response can help prevent obesity by promoting the production of hormones like corticotropin-releasing hormone (CRH). This hormone bioprocessing inhibits obesity by regulating the production of CRH, which is responsible for controlling stress levels in the body, and the receptor complex can be used to identify potential anti-obesity receptors such as the Neuropeptide Y (NPY) receptor for researching new effective obesity drugs to treat obesity. NPY receptors are a family of receptors belonging to class A G-protein coupled receptors, and they are activated by the closely related peptide hormones neuropeptide Y; these receptors are involved in the control of a diverse set of behavioral processes, including appetite [55,56,57,58]. KEGG pathway analysis indicated that the active saponins from PG could combat obesity through several pathways, including the PI3K-Akt signaling pathway, the Calcium, and the MAPK signaling pathway. In the PI3K-Akt signaling pathway, PI3K regulates the growth and differentiation of various cells, autophagy, and metabolism, while AKT is downstream in the pathway to modulate glucose and fat metabolism to combat obesity. The MAPK signaling pathway can combat obesity by regulating chemicals such as saponin [47,59].

The results of molecular docking expressed that the binding affinities of the PG active saponins and anti-obesity-related proteins selected for this study were all lower than −6.0, indicating that all the saponins and anti-obesity-related targets can be bound to fight against obesity [60]. This study chose to demonstrate the binding of dimethyl 2-O-methyl-3-O-a-D-glucopyranosyl platycogenate A and IL2, prosapogenin D and STAT3, 3″-O-acetylplatyconic acid A and PPARG, platycodin A and EGFR, platycodin D2 and HSP90AA1, deapioplatycoside E and GSK3B, platycodin D3 and JAK2, deapio-platycodin D3 and JUN, platycodin C and MTOR, and platycodin D and EGFR in the 3D and 2D results of the ten pairings containing all selected saponins and central anti-obesity targets to illustrate the rationale for this study.

The ADMET analysis indicated that all five selected active saponins showed quality biocompatibility in terms of toxicity prediction of the active saponins based on different toxicity model calculations without hepatotoxicity, carcinogenicity, and mutagenicity. Therefore, only compounds with an active toxicity model and a confidence level greater than 0.7 were considered [61]. 3″-O-acetylplatyconic acid A, platycodin A, and platycodin D2 need to be considered for toxicity by cytotoxicity in subsequent studies. After ADMET analysis, prosapogenin D appeared to be the least toxic of the top five active saponins and may have better outcomes in the treatment of obesity, making it more relevant for development as a novel anti-obesity drug.

Prosapogenin D could also be selected for experimental validation in subsequent studies. In the KEGG signaling pathway analysis of prosapogenin D, the PI3K-Akt signaling pathway was the biggest prominent anti-obesity signaling pathway. In another study, prosapogenin D treats and reduces obesity by improving the kinase pathway of action [17]. This also proves the veracity that prosapogenin D and other active saponins from PG can treat obesity. The results of prosapogenin D expressed that the binding energies of saponin prosapogenin D shortlisted by ADMET analysis with the central targets of the significant anti-obesity pathway were all less than −8.0, indicating that prosapogenin D has a better anti-obesity effect through the anti-obesity pathway and confirming the rationality of the experiment [60]. Prosapogenin D can be used as a representative active saponin compound for further studies and experimental validation. All molecular docking 3D and 2D results were also used to illustrate the fundamentals of network pharmacology studies.

## 4. Materials and Methods

### 4.1. Using Databases to Collect Active Compounds of PG Saponins

To screen for biologically active compounds, the Traditional Chinese Medicine Systems Pharmacology Database and Analysis Platform (TCMSP Version 2.3, https://old.tcmsp-e.com/tcmsp.php, accessed on 10 August 2023) was used to obtain the plant’s active compounds of PG saponins [31]. The TCMSP database contains information on the absorption, distribution, metabolism, and excretion (ADME) of each compound [62]. In TCMSP, the term “OB” stands for oral bioavailability. It refers to the speed and scope to which the active ingredient extracted from the herbal medicine is absorbed from the therapeutic product associated with the target. On the other hand, “DL” refers to drug-likeness, which is a qualitative parameter necessary for the design of new drugs. During the screening process, it is important to collect the active compounds of PG saponins that have both OB ≥ 30% and DL ≥ 0.18. These saponins should be used for subsequent target prediction [63].

### 4.2. The Intersection of PG Saponins and Obesity-Related Targets

The option to use the “Related Targets” feature of the TCMSP was used to collect targets associated with active components. Subsequently, conversion of protein names to corresponding gene names using the Uniprot database (www.uniprot.org/, accessed on 12 August 2023) and targets lacking corresponding gene names was excluded [64]. The resulting list comprises the targets of active saponin components within PG.

The databases of Online Mendelian Inheritance in Man (OMIM; www.omim.org/, accessed on 12 August 2023), Drugbank (www.drugbank.ca/, accessed on August 12, 2023), DisGeNet (www.disgenet.org/, accessed on 12 August 2023), and GeneCards (www.genecards.org/, accessed on 12 August 2023) were systematically searched using the keywords “obesity” and “insulin resistance.” The targets associated with obesity were linked to the targets of the active saponin components of PG to identify overlapping targets [33,65,66,67]. These intersections may be the focus of PG saponins in the fight against obesity.

### 4.3. Screening of Key Components and Targets of PG Saponins for Anti-Obesity

Imported into the Search Tool for the Retrieval of Interacting Genes/Proteins (STRING) database (https://string-db.org/, accessed on 12 August 2023) were potential targets, with choosing the target species to “Homo sapiens”. PPIs in the targets were examined, and the results were formatted in degree values. The data were formatted and then imported into Cytoscape 3.7.1 (https://cytoscape.org/, accessed on 12 August 2023) for presentation and processing, and then the “Network Analyzer” analyzed the PPI network.

### 4.4. Protein–Protein Interaction Analysis

Importing potential targets for anti-obesity into STRING, with choosing the target species to Homo sapiens, the protein–protein interactions (PPI) were analyzed for these targets [35]. Analytical results were shown and subsequently brought into Cytoscape 3.7.1 (https://cytoscape.org/, version 3.9.0, Boston, MA, USA, accessed on 12 August 2023) for presentation and processing. The PPI network was analyzed using the “Network Analyzer” and used the degree value to screen the core targets [36].

### 4.5. Enrichment Analyses of Potential Anti-Obesity Targets

In this research, there are enrichment analyses for potential targets were conducted by using the database for annotation, visualization, and integrated discovery (DAVID) (https://david.ncifcrf.gov/ (accessed on 23 August 2023)) and Metascape (http://metascape.org/gp/ (accessed on 25 August 2023)) and then using Origin 2018 software (http://originlab.com/ (accessed on 26 August 2023)) to complete visualization of results [68,69].

### 4.6. Saponin Compound-Anti-Obesity Target Network Construction

The objective of this research was to show the mechanism through which the active saponin ingredients of PG enhance anti-obesity. A network map of active component–core target pathway was constructed using Cytoscape 3.7.1 (https://cytoscape.org/, version 3.9.0, Boston, MA, USA, accessed on 12 August 2023) [36]. The active saponin components of PG, core targets, and signaling pathways of anti-obesity are all shown in this map.

### 4.7. Enrichment Analysis

Functional enrichment analysis using the DAVID database for annotation, visualization, and integrated discovery (Version 6.8; https://david.ncifcrf.gov/, accessed on 15 August 2023) [70,71,72] was conducted for both Gene Ontology (GO) and Kyoto Encyclopedia of Genes and Genomes (KEGG) pathways. The three types of GO terms, namely cellular component (CC), biological process (BP), and molecular function (MF), were employed. Bubble plots illustrating bioprocesses and pathways were generated by uploading the data to the Bioinformatics platform (http://www.bioinformatics.com.cn/ (accessed on 30 August 2023)). Statistical significance was determined using the classical hypergeometric test, and an adjusted *p*-value < 0.05 was considered significant [73].

### 4.8. Molecular Docking of PG Saponins and Potential Anti-Obesity Targets

The active compound structures of PG saponins were obtained in Spatial Data File (SDF) format from the NCBI PubChem online database (https://pubchem.ncbi.nlm.nih.gov/ (accessed on 3 September 2023)). Three-dimensional (3D) structures were generated using BIOVIA Discovery Studio Visualizer 2021 and saved in PDB format. Protein crystal structures of potential targets (EGFR, STAT3, JUN, GSK3B, PPARG, HSP90AA1, MTOR, IL2, and JAK2) were retrieved in PDB format from the Protein Data Bank (https://www.rcsb.org/ (accessed on 3 September 2023)) [74]. Ligands and water molecules were extracted from the crystal structure complex using BIOVIA Discovery Studio Visualizer 2021 Software. The grid was designed, and protein preparation was performed using the same software [75]. The proteins in PDB format were then imported into AutoDock Vina (version 1.2.0.), followed by uploading the key active compounds in PDB format. Conversion to pdbqt format was performed for both proteins and key active compounds using AutoDock Vina (version 1.2.0.). Subsequently, scriptwriting for molecular docking was carried out with AutoDock Vina (version 1.2.0.) using proteins and key active compounds in pdbqt format. The resulting docked complexes were visualized to assess the binding ability of molecules and targets using BIOVIA Discovery Studio Visualizer 2021 software [75]. A binding energy < 0 suggests the potential for spontaneous binding of a ligand to the receptor. The lower the energy score of the ligand and receptor binding configuration, the higher the likelihood of binding occurrence [76].

### 4.9. ADMET Analysis of PG Saponins

To assess the pharmacological similarity of these compounds, the absorption, distribution, metabolism, excretion, and toxicity (ADMET) indices associated with PG active saponin constituents were investigated. SMILES of PG active saponin constituent substances were collected using the PubChem database, followed using the SwissADME database (SwissADME Version 2023, http://www.swissadme.ch, accessed on 22 November 2023) and the ProTox-II database (ProTox-II Version 2021, https://tox-new.charite.de/protox_II/, accessed on 22 November 2023) for ADMET analysis [77,78].

## 5. Conclusions

This study explains and illustrates the saponins and molecular mechanisms that are positive in the treatment of obesity by a kind of plant-derived food PG. This study identified 18 active saponins and 44 core anti-obesity targets derived from PG using a network pharmacology approach. The study also identified three key pathways that may be involved in the anti-obesity mechanism of PG, namely the PI3K-Akt signaling pathway, the Calcium signaling pathway, and the MAPK signaling pathway, and the results suggest that the anti-obesity effect of active saponins from PG is a combined action through multiple targets and multiple pathways. Molecular docking results indicated that the major active saponins in PG could bind to and exert their effects on key anti-obesity protein targets in the pathway. Prosapogenin D was also screened for toxicity by ADMET analysis for KEGG analysis and molecular docking with regulatory proteins of key signaling pathways, and the results further demonstrated that PG-active saponins can play a role in anti-obesity. This study is based on the results of network pharmacological research and molecular docking. More animal experiments and clinical trials still need to be conducted to verify the underlying molecular mechanism of *Platycodon grandiflorum* saponin in vivo. The findings laid the foundation for additional research on anti-obesity saponins in PG and the mechanism of PG anti-obesity and in the development of plant-derived food.

## Figures and Tables

**Table 1:** A total of 18 saponin constituents in PG after the literature and database screening.

PubChem ID	Saponins	Molecular Formula	Structure	References
46173910	Platycodin A	C_59_H_94_O_29_	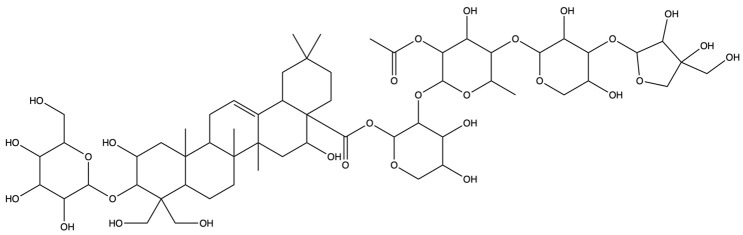	[27]
46173919	Platycodin C	C_59_H_94_O_29_	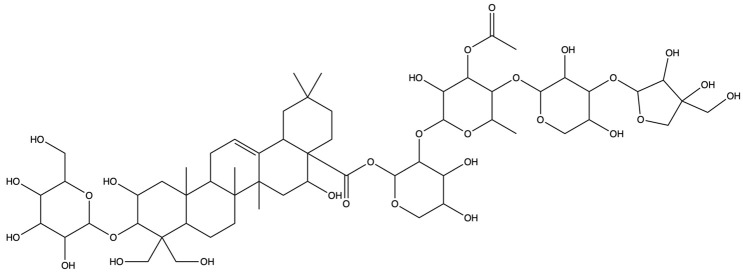	[27]
162859	Platycodin D	C_57_H_92_O_28_	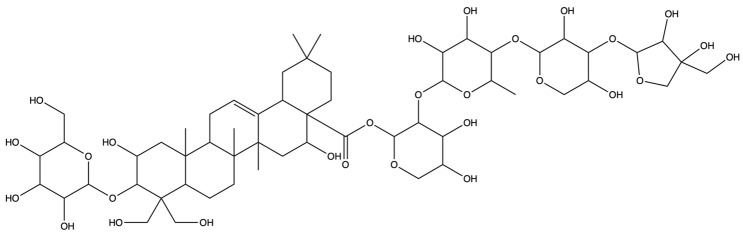	[27]
70698266	Deapioplatycodin D	C_52_H_84_O_24_	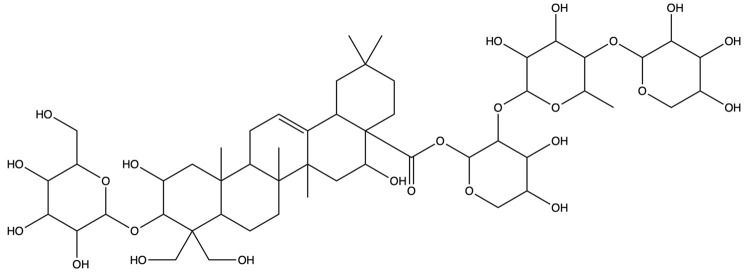	[27]
53317652	Platycodin D2	C_63_H_102_O_33_	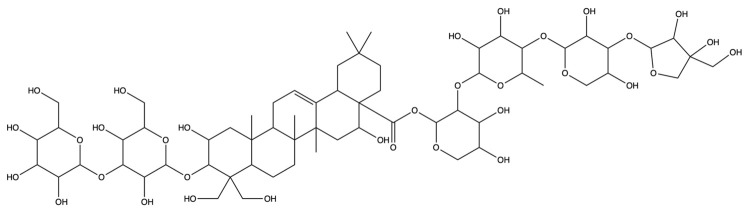	[27]
75251137	Platycodin D3	C_63_H_102_O_33_	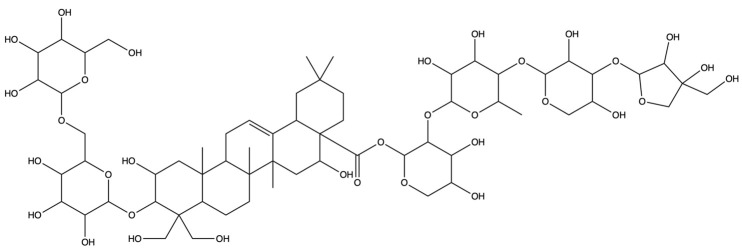	[27]
/	Deapioplatycodin D3	C_58_H_94_O_29_	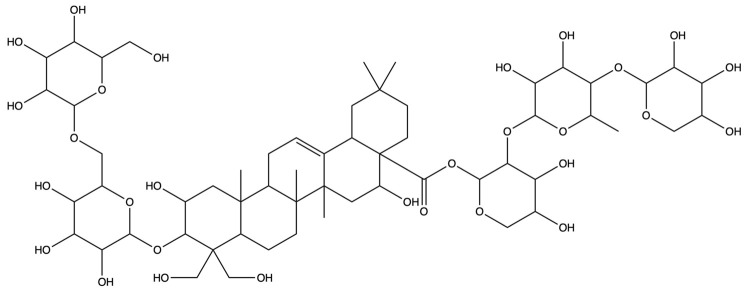	[27]
70698289	Deapioplatycoside E	C_64_H_104_O_34_	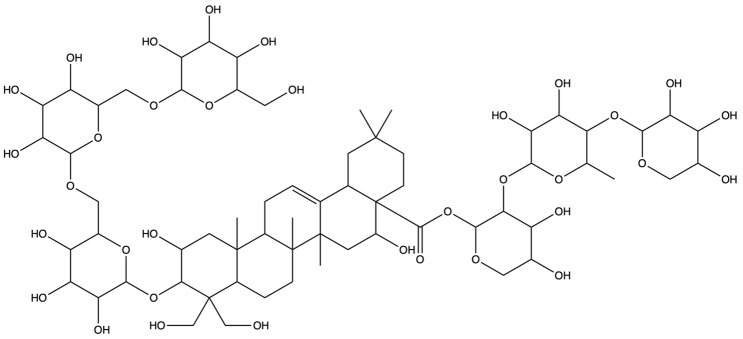	[28]
50900852	Platyconic acid B lactone	C_63_H_98_O_33_	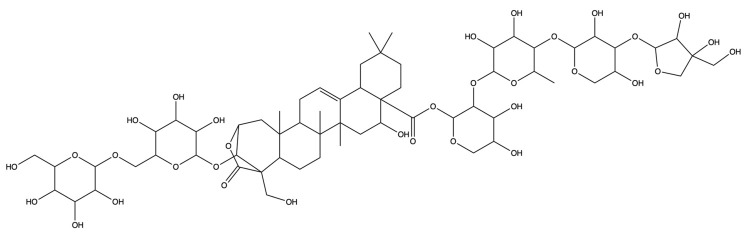	[28]
/	3″-O-acetylplatyconic acid A	C_59_H_92_O_30_	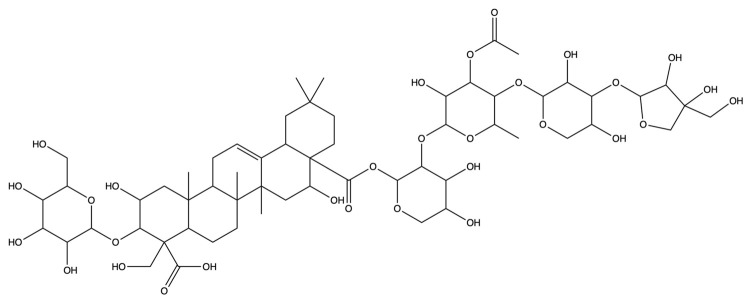	[28]
/	Prosapogenin D	C_36_H_58_O_8_	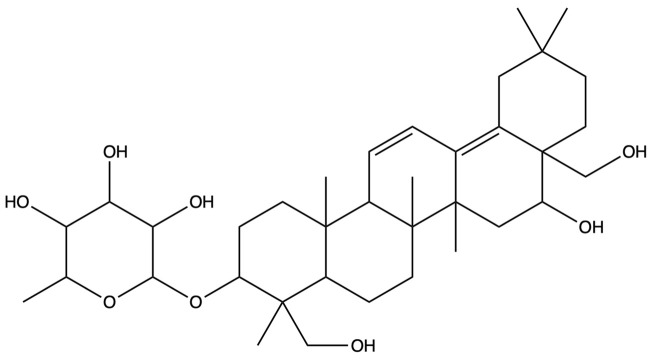	[29]
96023791	Polygalacin D	C_57_H_92_O_27_	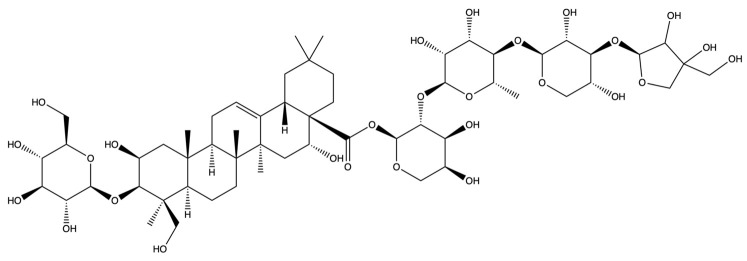	[28]
385678073	Polygalacin D3	C_63_H_102_O_32_	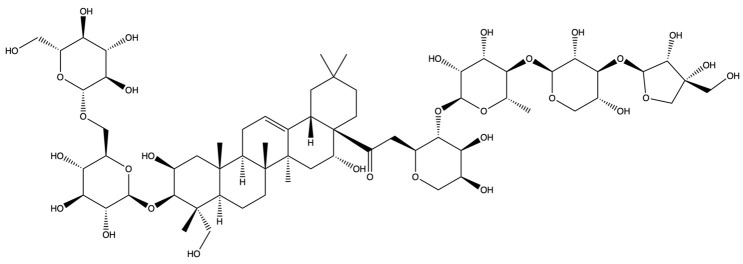	[28]
70698202	Platycoside E	C_69_H_112_O_38_	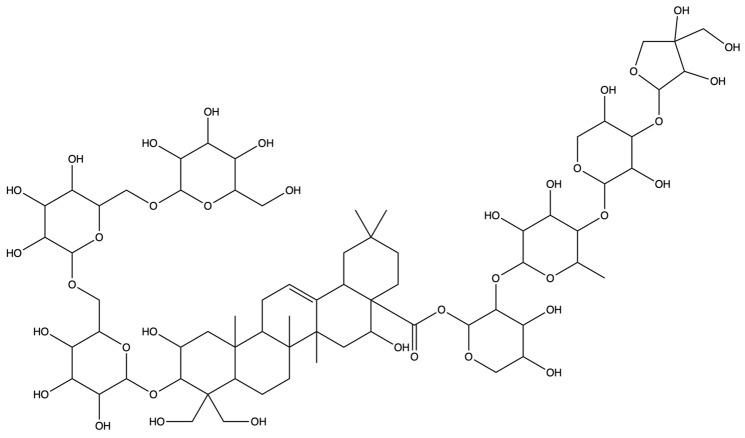	[28]
385678065	Polygalacin D2	C_63_H_102_O_32_	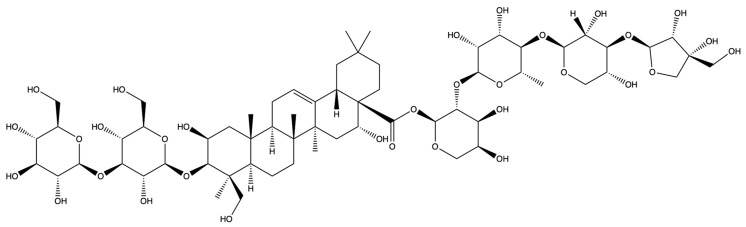	[28]
/	Dimethyl 2-O-methyl-3-O-a-D-glucopyranosyl platycogenate A	/	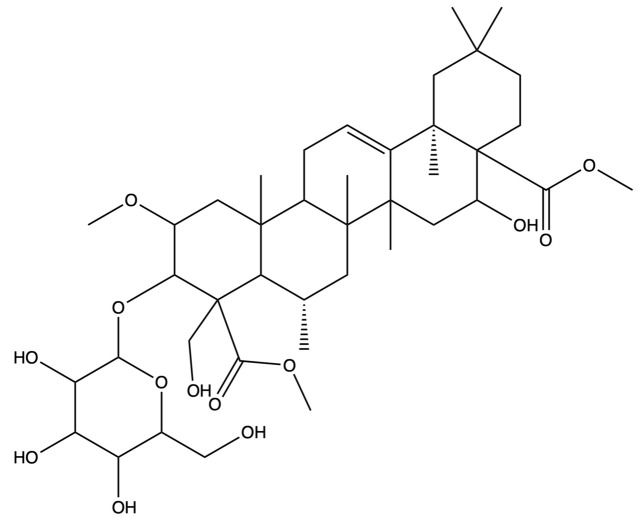	TCMSP
/	Platycodin V	/	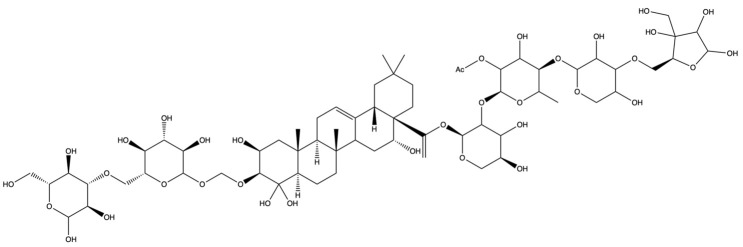	[27]

**Table 2:** ADMET analysis of top 5 saponins.

Saponins	Dimethyl 2-O-methyl-3-O-a-D-glucopyranosyl Platycogenate A	Prosapogenin D	3″-O-acetylplatyconic Acid A	Platycodin A	Platycodin D2
GI absorption	Low	Low	Low	Low	/
BBB permeant	No	No	No	No	/
P-gp substrate	Yes	Yes	Yes	Yes	/
CYP1A2 inhibitor	No	No	No	No	/
CYP2C9 inhibitor	No	No	No	No	/
CYP2C19 inhibitor	No	No	No	No	/
CYP2D6 inhibitor	No	No	No	No	/
CYP3A4 inhibitor	No	No	No	No	/
Log Kp (Skin permeation)	−8.29 cm/s	−7.14 cm/s	−16.40 cm/s	−16.26 cm/s	/
Predicted LD50	1500 mg/kg	4000 mg/kg	4000 mg/kg	4000 mg/kg	4000 mg/kg
Class	4	5	5	5	5
Hepatotoxicity	Inactive	Inactive	Inactive	Inactive	Inactive
Carcinogenicity	Inactive	Inactive	Inactive	Inactive	Inactive
Mutagenicity	Inactive	Inactive	Inactive	Inactive	Inactive
Cytotoxicity	Inactive	Inactive	Active (0.70)	Active (0.70)	Active (0.70)

**Table 3:** Molecular docking results of Prosapogenin D.

Protein	PDB ID	Binding Energy	Interaction
PI3K	4FA6	−8.139	Conventional Hydrogen Bond Carbon Hydrogen Bond Alkyl
AKT	4GV1	−8.494	Conventional Hydrogen Bond Carbon Hydrogen Bond Alkyl Pi-Alkyl

## Data Availability

The data presented in this study are available on request from the corresponding author.
